# Comprehensive Phytochemical Analysis and Evaluation of Antioxidant, Antimicrobial, Cytotoxic, and Immunomodulatory Activities of Commercial Cinnamon Bark Essential Oil (*Cinnamomum zeylanicum* L.)

**DOI:** 10.3390/ijms26136482

**Published:** 2025-07-05

**Authors:** Milja Živković, Isidora Stanisavljević, Nevena Gajović, Slađana Pavlović, Bojana Simović Marković, Ivan P. Jovanović, Snežana Cupara, Vanja Tadić, Ana Žugić, Marina T. Milenković, Ana Barjaktarević

**Affiliations:** 1 Department of Pharmacy, Faculty of Medical Sciences, University of Kragujevac, Svetozara Markovica 69, 34000 Kragujevac, Serbia; zivkovicmilja@gmail.com (M.Ž.); snezanacupara@yahoo.com (S.C.); 2 Center for Molecular Medicine and Stem Cell Research, Faculty of Medical Sciences, University of Kragujevac, Svetozara Markovića 69, 34000 Kragujevac, Serbia; isidorastanisavljevic97@gmail.com (I.S.); gajovicnevena@yahoo.com (N.G.); sladjadile@gmail.com (S.P.); bojana.simovic@gmail.com (B.S.M.); ivanjovanovic77@gmail.com (I.P.J.); 3 Department for Pharmaceutical Research and Development, Institute for Medicinal Plant Research “Dr. Josif Pančić”, Tadeuša Koscuška 1, 11000 Belgrade, Serbia; vtadic@mocbilja.rs (V.T.); azugic@mocbilja.rs (A.Ž.); 4 Department of Microbiology and Immunology, Faculty of Pharmacy, University of Belgrade, Vojvode Stepe 450, 11221 Belgrade, Serbia; marina.milenkovic@pharmacy.bg.ac.rs

**Keywords:** *Cinnamomum zeylanicum*, bark essential oil, antioxidant, antimicrobial, antitumor, immunomodulatory

## Abstract

The essential oil derived from the bark of *Cinnamomum zeylanicum* L., *Lauraceae*, has gained significant attention because of its numerous biological benefits. This study aimed to perform a phytochemical analysis of commercially available *Cinnamomum zeylanicum* bark essential oil and to evaluate its antioxidant, antimicrobial, immunomodulatory, and antitumor properties. GC–MS analysis was employed to determine the phytochemical composition. The major component of the total essential oil composition was (*E*)-cinnamaldehyde, constituting 77.93%, followed by eugenol (4.34%), *E*-caryophyllene (3.68%), and linalool (2.79%). The antioxidant activity was confirmed by DPPH, ABTS, CUPRAC, and TAC assays. In the broth microdilution assay, cinnamon essential oil demonstrated strong antimicrobial activity, with MIC values ranging from 7.37 to 29.50 µg/mL. Furthermore, cinnamon essential oil demonstrated selective antitumor activity by inducing apoptosis and cell-cycle arrest in human colorectal cancer cells (HCT116) while sparing non-cancerous cells (MRC-5). In HCT116 cells, cinnamon essential oil induced apoptosis, downregulated Cyclin D and p-AKT, and caused G1-phase arrest. Additionally, cinnamon essential oil modulated immune responses by reducing pro-inflammatory cytokine production in activated splenocytes and enhancing pro-inflammatory activity in naïve cells. These findings highlight the great potential of the cinnamon bark essential oil in the development of new therapeutic agents.

## 1. Introduction

Cinnamon (*Cinnamomum verum* J. Presl. or *Cinnamomum zeylanicum* L., *Lauraceae*), commonly known as ‘true cinnamon,’ is an aromatic spice that has been traditionally used for its medicinal and culinary properties [1]. Over the past twenty years, there has been a growing tendency to develop new herbal medicines from natural sources [2]. Recently, the essential oil derived from cinnamon bark has gained significant attention for its diverse biological activities, including antioxidant, antimicrobial, immunomodulatory, and anticancer effects [3]. These properties are largely attributed to the unique chemical composition of the essential oil, particularly the presence of bioactive compounds such as cinnamaldehyde, eugenol, cinnamyl alcohol, linalool, and various terpenoids, which play crucial roles in inhibiting harmful microorganisms, neutralising oxidative stress, modulating the immune response, and suppressing tumor development [1,3]. Cinnamaldehyde was found to be a major bioactive compound, making up 60–80% of the cinnamon bark essential oil (CEO) [4,5]. The specific composition of the oil can vary depending on factors such as the plant’s geographical origin and the method of extraction. Particularly, cinnamaldehyde, as a major component, is responsible for the antioxidant and antimicrobial properties of essential oil, among other effects [4,6]. The mechanism behind the CEO’s antioxidant activity is mainly described as its ability to neutralise free radicals and reduce oxidative stress [7]. Previous studies have shown that cinnamaldehyde and eugenol play crucial roles in inhibition of lipid peroxidation and protection against oxidative damage [3,7]. A study conducted by Denkova-Kostova R et al. [8] showed the efficacy of bark CEO as a natural antioxidant and antimicrobial agent. The oil demonstrated high antimicrobial activity against spore-forming bacteria, saprophytic microorganisms, fungi, and yeast. The essential oil was more effective against Gram-positive bacteria, compared to Gram-negative bacteria, because of the evident difference in the structure of the bacterial cell wall [8]. Additionally, CEO has demonstrated efficacy against drug-resistant strains of *Escherichia coli*, *Staphylococcus aureus*, and *Candida albicans* [9,10]. Along with its antioxidant and antimicrobial properties, CEO has been found to possess immunomodulatory and antitumor activity. Recent research indicates that compounds like cinnamaldehyde can modulate the immune response by regulating the production of pro-inflammatory cytokines and reducing inflammation. This makes cinnamon oil a promising agent for managing immune-related conditions, such as autoimmune diseases and chronic inflammatory disorders [11]. Previously published studies have reported the cytotoxic activity of CEO on several human and animal cancer cell lines, including lung cancer, breast adenocarcinoma, chronic myelogenous erythroleukemia, mouse breast cancer, and mouse lymphocytic leukemia cell lines [12,13]. It has been found that cinnamaldehyde induces apoptosis and inhibits the proliferation of cancer cells in various cancer models, including breast, lung, and colorectal cancers [14].

The present study aimed to perform a phytochemical analysis of commercially available *C. zeylanicum* bark essential oil and to expand the existing knowledge of its antioxidant, antimicrobial, immunomodulatory, and antitumor properties.

## 2. Results

### 2.1. GC–MS Analysis of the CEO

The gas chromatography–mass spectrometry (GC–MS) analysis of the bark CEO identified 27 constituents, which together represented 99.66% of the essential oil composition (Table 1). All identified components have been categorized into five categories: monoterpene hydrocarbons, oxygenated monoterpenes, sesquiterpenes, aromatic compounds, and cinnamic derivatives. A quantity of nine components within the monoterpene hydrocarbons category severally constituted 0.05–1.45%. Linalool was the most abundant component among the oxygenated monoterpenes (2.79%); *E*-caryophyllene (3.68%) among the sesquiterpene hydrocarbons, and eugenol (4.34%) among the aromatic compounds. However, the dominant component of the total essential oil composition was (*E*)-cinnamaldehyde, within the cinnamic derivatives category, with a share of 77.93%.

### 2.2. Antioxidant Activity

In this study, the antioxidant activity of *C. zeylanicum* bark essential oil was assessed using four different in vitro assays: the DPPH Radical Scavenging Activity Assay (DPPH), ABTS Radical Scavenging Activity Assay (ABTS), Cupric Ion Reducing Antioxidant Capacity Assay (CUPRAC), and Total Antioxidant Capacity Assay (TAC) assays. The results of antioxidant activity are presented in Table 2. According to IC_50_ values determined using the DPPH, ABTS, and CUPRAC assays (IC_50_ = 9.53 ± 0.2 mg/mL; 0.35 ± 0.01 mg/mL; and 0.14 ± 0.002 mg/mL, respectively), CEO has shown a weak antioxidant activity compared to BHT (IC_50_ = 0.011 mg/mL; 0.006 mg/mL; and 0.02 mg/mL, respectively). The results of the TAC assay showed a value of 0.01 mg AAE/g for CEO, compared to 9.80 mg AAE/g for BHT (Butylated hydroxytoluene). This indicates that the CEO has a low capacity with respect to the reduction of molybdenum ions (Mo^+6^ into Mo^+5^).

### 2.3. Antimicrobial Activity

The in vitro antimicrobial activity of the cinnamon bark essential oil was evaluated using the broth microdilution method, and according to the CLSI recommendations [15].

The obtained MIC values for CEO were in the range of 7.37–29.50 µg/mL (Table 3). The most pronounced antimicrobial activity was observed against *S. aureus*, *S. epidermidis*, and *C. albicans*, with identical MIC values of 7.37 µg/mL. The CEO was less active against *B. subtilis*, *E. coli*, *K. pneumoniae*, and *S. enterica* serovar Abony, with a MIC value of 14.75 µg/mL. However, *P. aeruginosa* was the most resistant to CEO (MIC = 29.50 µg/mL). According to the results presented in Table 3, Gram-positive bacteria, except *B. subtilis*, were more susceptible to CEO, compared to the tested Gram-negative bacteria. Generally, all MIC values for tested microbial strains are very low (significantly lower than 100 µg/mL), and that fact can classify CEO in the category of essential oils with strong antimicrobial activity. However, the observed MIC values of CEO were notably higher than those for the standard antibiotics and the antifungal agent, indicating lower antibacterial potency compared to synthetic agents.

### 2.4. Evaluation of In Vitro Cytotoxicity of CEO

**Figure 1:**
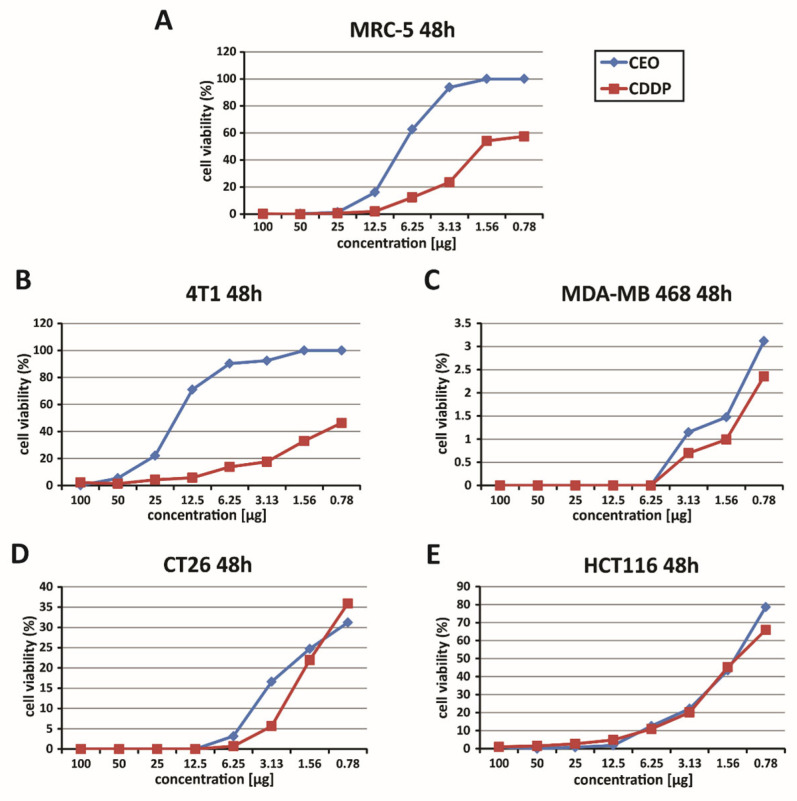
Dose-dependent cytotoxic effects of CEO. This figure illustrates the dose-dependent cytotoxicity of the CEO on various cell lines, as assessed by the MTT assay, following 48 h of exposure. The graphs show the survival rates of human fibroblasts (MRC-5) (**A**), murine and human breast carcinoma cells (4T1 and MDA-MB 468) (**B**,**C**), and murine and human colon cancer cells (CT26 and HCT116) (**D**,**E**), treated with the CEO and cisplatin (CDDP). The data presented represent mean values from three independent experiments, each conducted in triplicate.

The cytotoxic effects of the CEO were assessed using the MTT assay. This analysis was performed on several cell lines, including mouse (4T1) and human (MDA-MB-468) breast cancer cells and mouse (CT26) and human (HCT116) colon cancer cells, as well as the non-cancerous human fibroblast cell line (MRC-5). The cell lines were exposed to varying concentrations of the CEO (ranging from 100 to 0.78 µg) for 48 h. The findings indicate that the CEO significantly affected the viability of the 4T1, MDA-MB-468, HCT116, and CT26 cancer cell lines (Figure 1), while having minimal impact on the non-cancerous MRC-5 cells (Figure 1). The CEO was shown to reduce MRC-5 cell viability in a dose-dependent manner (Figure 1). However, at concentrations ranging from 0.78 to 100 µg, CEO exhibited a lower cytotoxic effect on MRC-5 cells, compared to its effects on cancerous cell lines (4T1, MDA-MB-468, HCT116, and CT26). Furthermore, cisplatin (*cis*-diamminedichloroplatinum(II), CDDP) exhibited stronger cytotoxic effects on mouse 4T1 and CT26 cell lines, as well as human MDA-MB 468 and HCT116 cell lines, in comparison to CEO, under the same experimental conditions. It also reduced the viability of non-cancerous MRC-5 cells more than did CEO (Figure 1).

In the context of the study of the cytotoxic effectiveness of CEO, a detailed assessment of the half-maximal inhibitory concentration (IC_50_) values was conducted. This analysis focused on breast cancer cell lines (mouse 4T1 and human MDA-MB-468) as well as colon cancer cell lines (mouse CT26 and human HCT116). The IC_50_ values, representing the concentration needed to inhibit 50% of cell viability, are listed in full in Table 4. Upon examining these values, it was found that the CEO demonstrated more potent cytotoxic effects against the human HCT116 cells, compared to other cell lines. Also, CDDP is one of the most commonly used chemotherapeutic agents and is known for its cytotoxic effects on both cancerous and non-cancerous cells. However, CEO exhibited stronger cytotoxicity toward HCT116 cells, compared to CDDP, while simultaneously showing lower toxicity towards non-cancerous MRC-5 cells, as shown in Table 4.

The therapeutic selectivity of the cinnamon essential oil was further assessed by calculating the selectivity index, which is presented in Table 5. One of the key findings of this study was the notably high selectivity index for the CEO, with the HCT116 cell line exhibiting the highest selectivity index among all tested cell lines. This suggests that CEO is significantly more cytotoxic to the HCT116 cancer cells than to other cancerous and non-cancerous cells. Moreover, although CDDP exhibited greater cytotoxicity, it displayed lower selectivity towards non-cancerous MRC-5 cells, confirming its stronger but less selective effect compared to CEO (Table 5). Based on these findings, further research was conducted using the HCT116 cell line.

### 2.5. Assessment of Apoptotic Cell Death Induced by CEO

**Figure 2:**
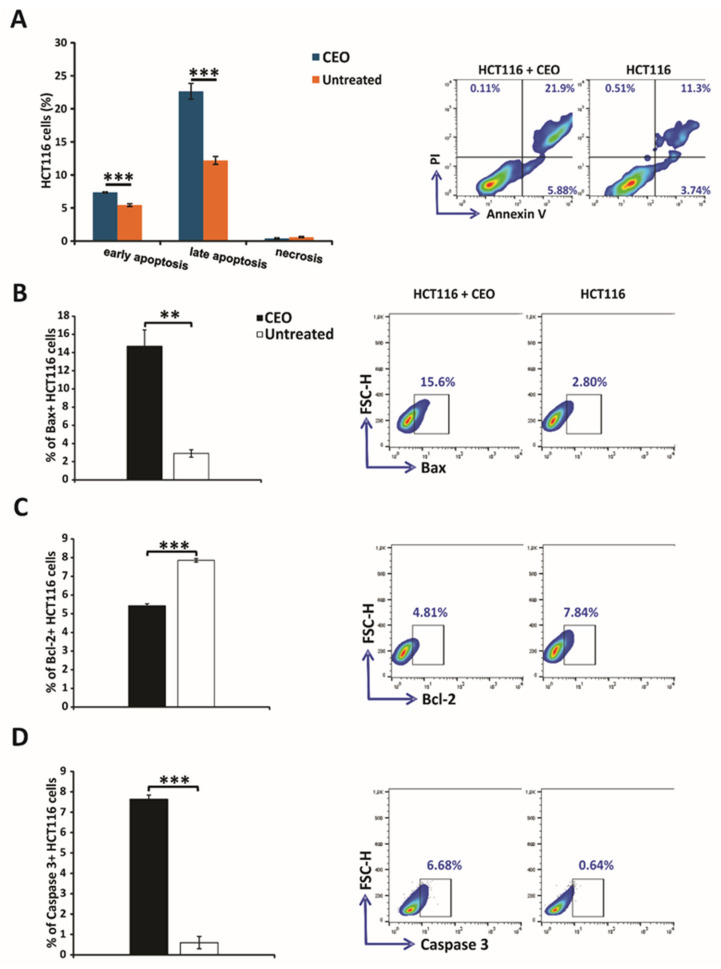
Induction of apoptosis and modulation of apoptosis-related proteins in HCT116 colorectal cancer cells by the CEO. Apoptosis was assessed using flow cytometry with Annexin V (FITC) and propidium iodide (PI) double staining to differentiate between viable (AnnV−PI−), early apoptotic (AnnV+PI−), late apoptotic (AnnV+PI+), and necrotic (AnnV−PI+) populations (**A**). Additionally, the expression levels of key apoptosis-related proteins, including Bax (**B**), Bcl-2 (**C**), and caspase-3 (**D**), were evaluated in both untreated and CEO-treated HCT116 cells after 24 h. Statistical analysis was performed using the non-parametric Mann–Whitney test. Statistical significance is indicated by ** *p* < 0.01, and *** *p* < 0.001, comparing CEO-treated cells to the untreated control group.

The evaluation of apoptosis in HCT116 cells treated with CEO was performed using the Annexin V and propidium iodide double-staining assay. In this analysis, cells were treated with the CEO and then stained with Annexin V-FITC and PI. The results, shown in Figure 2A, demonstrated that a significant portion of HCT116 cells underwent both early and late apoptosis after 24 h of treatment with the CEO. Notably, necrosis does not appear to be the mechanism of cell death through which the CEO exerts its effects, as HCT116 cells did not undergo necrosis (Figure 2A). These findings highlight the dual mechanism of the CEO, which promotes cell death via apoptotic pathways, by suggesting its potential as a powerful anti-carcinoma agent. The results of this study indicate that treatment with IC_50_ concentrations of the CEO significantly increased the percentage of Bax+ HCT116 cells, while simultaneously reducing the percentage of Bcl-2+ HCT116 cells (Figure 2B,C). Furthermore, the CEO was shown to enhance the percentage of caspase-3+ HCT116 cells (Figure 2D). The observed increase in caspase-3 expression, coupled with a decrease in Bcl-2 expression, points to potential mechanisms of action for the CEO, particularly in inducing apoptosis. Additionally, the marked increase in Bax, a pro-apoptotic protein, following treatment with the CEO, underscores the role of CEO in promoting apoptosis in HCT116 cells.

### 2.6. Effects of CEO on Cell Cycle and Proliferation in HCT116 Cancer Cells

**Figure 3:**
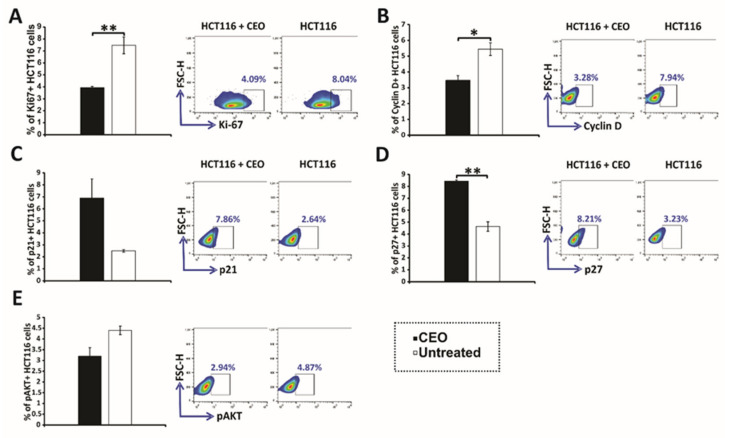
Expression of cell-cycle and signaling proteins in HCT116 cells treated with CEO. The expression of Ki67 (**A**), Cyclin D (**B**), p21 (**C**), p27 (**D**), and p-AKT (**E**) in HCT116 cells treated with the CEO is displayed. Representative FACS plots show the expression of key proteins following 24 h of exposure to the CEO. The data, obtained from flow cytometry, are presented as the mean ± SEM from three independent experiments. Statistical analysis was performed using the non-parametric Mann–Whitney test. Statistical significance is indicated by * *p* < 0.05 and ** *p* < 0.01, highlighting significant differences in protein expression between CEO-treated cells and the untreated control group.

In the investigation of CEO’s antiproliferative effects, Ki67 expression levels were analyzed in HCT116 cells. The results demonstrated a significant reduction in Ki67 expression in CEO-treated HCT116 cells compared to untreated control cells (Figure 3A), indicating a notable decrease in cell proliferation. To assess the effect of the CEO on Cyclin D, HCT116 cells were treated with an IC_50_ concentration of the essential oil for 24 h. The results, shown in Figure 3B, reveal that the CEO significantly decreased the percentage of Cyclin D^+^ cells, suggesting a reduction in the number of cells progressing from the G1 phase to the S phase. This selective downregulation of Cyclin D indicates that the CEO specifically interferes with cell-cycle progression at the G1 phase, potentially inducing cell-cycle arrest and preventing further proliferation of HCT116 cells.

Treatment with CEO induced significant alterations in the expression of key cell-cycle regulators in HCT116 cells. Specifically, there was an increase in the proportion of cells expressing the tumor suppressors p21 and p27 (Figure 3C,D). This upregulation is likely to contribute to CDK suppression, leading to cell-cycle arrest. Consistent with these findings, the increased expression of p21 and p27 was accompanied by a reduction in cyclin D levels, further reinforcing the disruption of cell-cycle progression. Additionally, CEO treatment led to a significant decrease in the levels of phosphorylated AKT (p-AKT) (Figure 3E). The reduction in p-AKT, a critical component of oncogenic signaling pathways that support cell survival and proliferation, suggests a potential antitumor effect of the CEO. These changes in p21, p27, and p-AKT expression highlight the CEO’s ability to disrupt key processes involved in cell-cycle regulation and survival signaling, underscoring its potential as an effective anticancer agent.

### 2.7. In Vitro, CEO Reduces the Concentration of Pro-Inflammatory Cytokines

**Figure 4:**
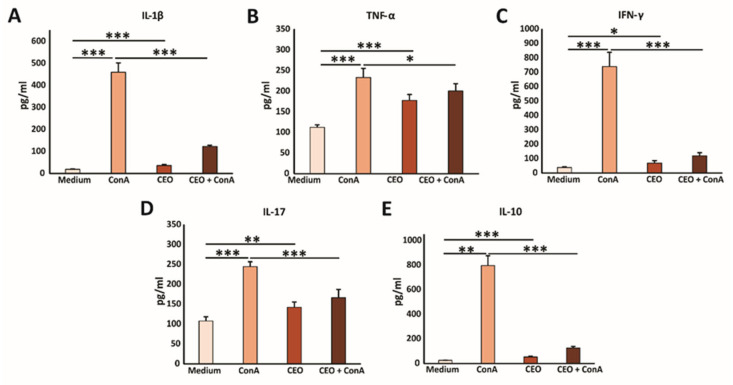
CEO decreases cytokine production in vitro. The graphs display the concentrations of IL-1β (**A**), TNF-α (**B**), IFN-γ (**C**), IL-17 (**D**), and IL-10 (**E**) in the supernatants of splenocytes from healthy mice after 24 h of incubation with medium only, ConA, CEO, or a combination of CEO and ConA. The data are presented as the mean ± SEM. Statistical analysis was performed using the non-parametric Kruskal–Wallis test. Statistical significance is indicated by * *p* < 0.05, ** *p* < 0.01, and *** *p* < 0.001, highlighting significant differences in cytokine concentration between groups.

**Figure 5:**
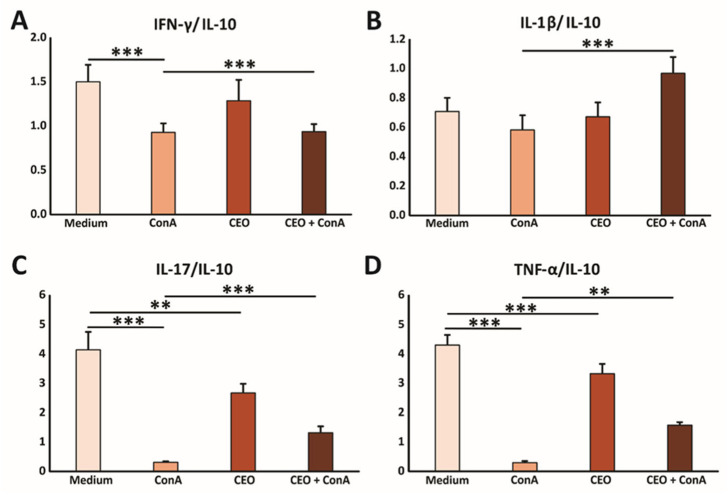
CEO facilitates the predominance of the pro-inflammatory cytokine milieu in vitro. The graphs display the ratios of IFN-γ/IL-10 (**A**), IL-1β/IL-10 (**B**), IL-17/IL-10 (**C**), and TNF-α/IL-10 (**D**) measured in the supernatants of splenocytes from healthy mice after 24 h of incubation with medium only, ConA, CEO, or a combination of CEO and ConA. The data are presented as mean ± SEM. Statistical analysis was performed using the non-parametric Kruskal–Wallis test. Statistical significance is indicated by ** *p* < 0.01, and *** *p* < 0.001, highlighting significant differences in cytokine concentration between groups.

In further research, the immunomodulatory effects of CEO were examined. Splenocytes freshly isolated from healthy mice were cultured with medium, ConA, CEO, or both CEO and ConA simultaneously. Treatment with ConA only, a poly-clonal T cell activator (21), notably elevated the levels of IL-1β, TNF-α, IFN-γ, IL-17, and IL-10 in the cell supernatant after 24 h, compared to untreated cells (Figure 4A–E). CEO by itself also significantly raised the concentrations of IL-1β, TNF-α, IFN-γ, IL-17, and IL-10, compared to splenocytes incubated in medium (Figure 4A–E). However, the combined treatment of ConA and CEO significantly lowered the levels of IL-1β, TNF-α, IFN-γ, IL-17, and IL-10, compared to ConA stimulation (Figure 4A–E). Furthermore, the CEO appears to promote a predominance of pro-inflammatory cytokines over IL-10, which is shown in Figure 5A–D. This effect is more pronounced in non-activated cells, and likely demonstrates components of the innate immune response. Interestingly, while CEO exerts an immunosuppressive effect on activated cells, it simultaneously activates naïve cells, thereby exhibiting a dual effect on immune-cell functionality.

## 3. Discussion

Plants provide an abundant source of potent medicinal compounds called phytochemicals, many of which play multiple roles in folk medicine worldwide. The application of different essential oils is associated with several biological properties, including antioxidant, antimicrobial, antiviral, anticancer, anti-inflammatory, immunomodulatory and antiprotozoal activities [16].

### 3.1. GC–MS Analysis

Generally, the data obtained by GC–MS analysis in this study were consistent with reports in the literature on the chemical composition of bark CEO. (*E*)-cinnamaldehyde is a dominant component of the bark CEO, present at a high concentration, according to several authors [8,10]. The concentrations of (*E*)-cinnamaldehyde in the bark CEO were in the range of 46–91.82%, depending on the plant’s habitat [17,18,19]. When comparing the results of this study with findings in the literature, we observed that our sample of CEO showed a higher concentration of (*E*)-cinnamaldehyde (77.93%) than the Iranian sample (71.5%), and the concentration reported by Elgendy et al. (64.84%) [18]. On the other hand, the concentration of cinnamaldehyde in the bark CEO obtained in this study was lower than in the Indian sample (91.82%) [19]. The other quantitatively most representative components in our CEO sample were as follows: eugenol (4.34%), (*E*)-caryophyllene (3.68), and linalool (2.79), respectively. Additionally, differences were evident in the levels of linalool and sesquiterpenes in the study conducted by Behbahani et al., while our study demonstrated greater concentrations of benzyl benzoate and other cinnamic derivatives [17]. (*E*)-cinnamaldehyde, as the major bioactive compound, is considered to be responsible for the biological activities of bark CEO, including the antimicrobial, antioxidant, and anti-inflammatory properties of the oil [1]. Its dominance suggests a central role in defining the overall activity of the CEO. The high percentage of (*E*)-cinnamaldehyde, combined with the contributions of oxygenated monoterpenes and eugenol, suggested significant potential applications in antimicrobial and antioxidant contexts.

Minor components, including monoterpene hydrocarbons (α-pinene, p-cymene, and limonene + β-phellandrene), and oxygenated monoterpenes (linalool and 1,8-cineole) contribute to the CEO’s bioactivity, particularly its antimicrobial and antioxidant effects [2,3]. Sesquiterpene hydrocarbons, namely, (*E*)-caryophyllene and α-humulene, add anti-inflammatory and analgesic properties [5].

### 3.2. Antioxidant Activity

It is well established that antioxidant activity cannot be accurately assessed using a single test; therefore, four commonly used assays, which differ in their underlying principles, have been employed [20]. The antioxidant activity of essential oils is likely due to the synergistic interactions among their constituents, with the major components playing a key role in this beneficial biological effect [17]. The antioxidant activity of (*E*)-cinnamaldehyde, α-pinene, eugenol, and β-caryophyllene has been reported in the literature [20,21,22]. However, based on the results of the DPPH, ABTS, and CUPRAC assays, the antioxidant activity of the CEO was weak, with an IC_50_ value higher than 100 µg/mL. The CEO sample was the most effective in reducing cupric ions, which was confirmed in the CUPRAC assay (IC_50_ = 0.14 ± 0.002 mg/mL). Our results were consistent with previous studies (Gheorghe-Irimia et al. and Elgamma et al.), which also found IC_50_ values above 0.1 mg/mL in the DPPH assay [9,23]. However, most studies reported the antioxidant activity of CEO as DPPH radical scavenging activity, expressed as the percent of inhibition, which prevents direct comparison with our results. These results ranged from 21.3% to 71.12% in DPPH inhibition [8,9,16,23,24]. Factors such as the plant’s geographical origin, plant parts used, and extraction methods may have influenced variations in chemical profile and, more directly, the antioxidant properties of the essential oil. The qualitative profiles of the CEO samples from different sources were similar, but quantitative differences were observed in the key bioactive compounds. Kallel et al. (2019) reported weak antioxidant activity (21.3% DPPH inhibition) for CEO, despite a cinnamaldehyde content determination similar to our sample (77.34% vs. 77.93%). In contrast to this study, our research confirmed the presence of compounds such as linalool and (*E*)-caryophyllene [25]. The classification of samples as moderate or strong antioxidants based on high percentages of DPPH radical inhibition was related to their higher contents of linalool (7.00%), *β*-caryophyllene (6.40%), and 1,8-cineole (5.40%), which enhance antioxidant activity via hydrogen donation [17]. Moreover, it was observed that CEO, with a high percentage of DPPH radical scavenging, which corresponds to high antioxidant activity, contains 83.71 ± 0.69% of *cis*-cinnamaldehyde, as its major compound. In our sample, the dominant compound was (*E*)-cinnamaldehyde (77.93%, Table 1), which is the *trans* stereoisomer of cinnamaldehyde. Differences between *cis* and *trans* isomers can influence the biological activity of essential oil, including its radical-scavenging ability. Additionally, the presence of terpinolene and D-limonene may have contributed to the higher antioxidant activity compared to our sample [8]. There is a lack of specific, comparable data on the antioxidant potential of CEO, as evaluated by the CUPRAC and ABTS assays, in the literature. The result obtained for CEO total antioxidant capacity, as determined by the TAC assay, was found to be 0.01 mg AAE/g (equivalent to 10 µg AAE/g). However, reports in the literature have determined higher values of CEO total antioxidant capacity (91.57 μg AAE/mL; 0.27 mg AAE/mg) [23,25]. These differing results highlight the importance of using multiple assays to obtain a comprehensive understanding of a sample’s antioxidant capacity.

In addition, the differences in the chemical composition and antioxidant activity of *C. zeylanicum* Blume could be attributed to the two different chemotypes identified by Farias et al. [26,27]. Chemotype differences significantly influence the functional properties of essential oils, and their potential applications in the pharmaceutical and food industries.

### 3.3. Antimicrobial Activity

The available in vitro and in vivo studies suggest that CEO exhibits broad-spectrum antimicrobial and antifungal activity. The suggested antimicrobial mechanism involves the ability of hydrophobic essential oils to disrupt the bacterial cell membrane and its structures [28]. Studies in the literature have proposed a classification of the plant materials, including the essential oils, which is based on MIC values. There are slight differences within the available literature data. Generally, essential oils with MIC values lower than 100 μg/mL are considered to be strong antimicrobials; those between 100 and 500 μg/mL are considered to be moderate inhibitors; and those between 500 and 1000 μg/mL are considered to be weak antimicrobial agents [28,29,30]. According to this classification, our sample of bark CEO expressed extremely strong antimicrobial activity against all tested microbial strains, with MIC values much lower than 100 µg/mL (7.37–29.50 µg/mL). Gram-negative bacteria, including *E. coli*, *K. pneumoniae*, *P. aeruginosa*, and *S. enterica* serovar Abony, have shown higher MIC values, indicating greater resistance. This can be associated with the additional layer of lipopolysaccharides (LPS) in their outer membrane, which serves as a barrier to the entry of many antimicrobial agents. Our finding of greater resistance of Gram-negative bacteria to essential oil was consistent with data in the literature [31,32,33]. The antimicrobial activity of CEO is well-documented in the literature. The study conducted by Elgammal et al. [23] confirmed the antimicrobial activity of bark CEO, using the agar well diffusion method, in line with our findings. They observed that bark CEO inhibits the growth of both Gram-positive and Gram-negative bacteria, with greater susceptibility observed in Gram-positive bacteria and fungi [23]. In tension with the results of Unlu et al. [34], our sample demonstrated greater antimicrobial activity, with significantly lower MIC values against all mutual microbial strains, although their study included a broader microbial spectrum [34]. Furthermore, our findings were supported by the results of Behbahani et al. [17], who reported the valuable antimicrobial activity of bark CEO, especially against Gram-positive bacteria. The comprehensive study of Saki et al. revealed the very potent antimicrobial effects of bark CEO against 150 extensively drug-resistant isolates, (60 Gram-positive and 90 Gram-negative bacteria strains) [35]. The low effective concentrations of CEO indicated the need for further investigations and highlight the great potential of CEO in the synthesis of new antimicrobial agents, particularly against multidrug-resistant bacteria [35]. However, the variations across studies may stem from differences in testing methods, extraction techniques, essential oil composition, and microbial strains.

According to the results of this study, the CEO exhibited lower antimicrobial activity compared to the standard antibiotics minocycline and amikacin and the antifungal agent nystatin. However, the great antimicrobial potential of CEO as a plant-derived product could be useful in adjuvating an antibiotic effect. There is evidence that a combination of CEO with colistine and ciprofloxacin demonstrated synergistic activity against multidrug resistant *P. aeruginosa* [36,37].

The antimicrobial activity of bark CEO is largely attributed to its chemical composition, particularly the high content of (*E*)-cinnamaldehyde (77.93%). Minor constituents, such as eugenol (4.34%) and *E*-caryophyllene (3.68%), further enhance this effect by disrupting microbial cell membranes and inhibiting vital enzymes.

### 3.4. In Vitro Evaluation of the Cytotoxic Effects of CEO

Nowadays, there is an increasing interest in exploring the antitumor and immunomodulatory activities of essential oils, which are primarily influenced by the major phytochemicals but also by the total composition of the oil, due to potential synergistic, additive, or antagonistic effects [38]. The findings of this study indicated that the CEO significantly affected the viability of the 4T1, MDA-MB-468, HCT116, and CT26 cancer cell lines, while having minimal impact on the non-cancerous MRC-5 cells. The IC_50_ values obtained for all evaluated cancer cell lines indicate that our CEO sample could be considered as a potential candidate in the development of anticancer drugs. This assessment is based on the criteria established by the U.S. National Cancer Institute, which recognizes plant-derived compounds with IC_50_ values below 30 μg/mL as potential anticancer agents. [12]. The cytotoxic effect of CEO against the human HCT116 cells was higher, when compared to cisplatin (CDDP), a widely used chemotherapeutic agent [39]. The antitumor effects of CEO were validated in numerous in vitro and in vivo studies [12,39,40]. In a study conducted by Nile et al. [41], an IC_50_ of 13.5 µg for cinnamon bark extract against human HCT116 cells was reported, while our study showed a significantly greater potency associated with CEO (IC_50_ = 1.18 µg). These differences might be attributed to the different cinnamon bark-derived products (extract vs. essential oil) and the varying concentrations of cinnamaldehyde [39]. Moreover, CEO has shown anticancer potential against several human cancer cell lines, including breast, lung, and prostate carcinomas [12,39].

Additionally, Kallel et al. [25] reported the pronounced cytotoxic effects of CEO against cervical cancer (HeLa) and Burkitt’s lymphoma (Raji) cell lines, with IC_50_ values below 1 μg/mL, further supporting its potential as an effective anticancer agent.

When developing low-toxicity drugs, it is essential to assess their anti-proliferative effects on normal, non-cancerous cells, such as the MRC-5 cell line. Our results showed a dose-dependent reduction of MRC-5 cell viability, and a lower cytotoxic effect of CEO on MRC-5 cells compared to its effects on cancerous cell lines (4T1, MDA-MB-468, HCT116, and CT26). This selective reduction in cytotoxicity toward MRC-5, combined with its significant impact on tumor cell viability, suggested that the CEO may offer selective cytotoxicity in targeting cancer cells. Our findings were consistent with previous studies demonstrating the anti-proliferative effects of cinnamon bark on normal cells, including CCD-112CoN normal colon cells and primary mouse lymphocytes [42]. One of the key findings of this study was the notably high selectivity index for the CEO, with the HCT116 cell line exhibiting the highest selectivity index among all tested cell lines. This suggested that the CEO exhibited pronounced cytotoxicity toward HCT116 cancer cells, along with strong selectivity over other cancerous and healthy cells, highlighting its potential for targeted cancer therapy. These findings prompted further research on the HCT116 cell line.

These findings expand the existing knowledge of CEO’s antitumor activity and support further investigations into its potential as an anticancer drug.

### 3.5. Assessment of Apoptotic Cell Death Induced by CEO

Anexin V FITC staining, which occurs before membrane integrity is lost, indicates early apoptotic cells as Annexin V FITC positive and PI negative; viable cells as both Annexin V FITC negative and PI negative; and late apoptotic or dead cells as both Annexin V FITC positive and PI positive [43]. The results of this study demonstrated that a significant portion of HCT116 cells underwent both early and late apoptosis after 24 h of treatment with the CEO. Notably, necrosis does not appear to be the mechanism of cell death through which the CEO exerts its effects, as HCT116 cells did not undergo necrosis. These findings highlight the dual mechanism of the CEO, which promotes cell death via apoptotic pathways, suggesting its potential as a powerful anti-carcinoma agent. Kubatka et al. [13] reported similar findings in human adenocarcinoma cell lines (MDA-MB-231 and MCF-7) treated with CEO for 24 to 72 h. Significant increases in the proportions of MDA-MB-231 cells in early and late apoptotic stages were observed, whereas MCF-7 cells showed minimal presence in the late apoptotic or necrotic phase.

Apoptosis is mainly initiated through two pathways: the extrinsic pathway, which involves death receptors activating caspase-3, and the intrinsic or mitochondrial pathway [40]. The intrinsic apoptotic pathway is initiated by an imbalance between pro-apoptotic and anti-apoptotic proteins (e.g., Bax and Bcl-2), leading to mitochondrial membrane permeabilization, cytochrome c release, and subsequent activation of caspase-3, culminating in apoptosis [42]. Bax (Bcl-2-associated X protein), a pro-apoptotic member of the Bcl-2 protein family, induces cell death by enhancing the release of cytochrome c from the mitochondria [44]. Alternatively, Bcl-2 (B-cell lymphoma 2) acts as an anti-apoptotic protein that prevents apoptosis by inhibiting the activity of pro-apoptotic proteins [45]. Caspase-3, a key executioner caspase in the apoptosis process, is activated in apoptotic cells through both the extrinsic (death ligand) and intrinsic (mitochondrial) pathways. It plays a central role in cleaving several critical cellular proteins, driving the morphological and biochemical alterations that define apoptosis [46]. The analysis of apoptotic markers in this study indicated that treatment with IC_50_ concentrations of the CEO significantly increased the percentage of Bax+ HCT116 cells, while simultaneously reducing the percentage of Bcl-2+ HCT116 cells. Moreover, the treatment with CEO resulted in a higher percentage of caspase-3+ HCT116 cells. The observed increase in caspase-3 expression, coupled with a decrease in Bcl-2 expression, points to potential mechanisms of action for the CEO, particularly in inducing apoptosis. Additionally, the marked increase in Bax, a pro-apoptotic protein, following treatment with the CEO underscored its role in promoting apoptosis in HCT116 cells. According to Nile et al. [41], cinnamon extract induced apoptosis in HCT116 cells through activation of the caspase cascade. Moreover, there was growing evidence supporting the regulatory role of phytochemicals in modulating the Bax/Bcl-2/caspase-3 signaling pathway in breast cancer. A significant increase in the Bax/Bcl-2 ratio and caspase-3 expression observed in mammary tumors of rats treated with CEO, as reported by Kubatka et al. [13], aligned with our findings in HCT116 cells.

### 3.6. Effects of CEO on Cell Cycle and Proliferation in HCT116 Cancer Cells

Ki67 is a well-established proliferation marker, expressed in actively dividing cells (G1, S, G2, and M phases), but absent during the resting phase (G0) cell cycle. Its strong correlation with cell division makes it valuable for assessing tumor growth and evaluating anticancer therapy efficacy in both clinical and research settings [47]. The results of this study revealed a significant reduction in Ki67 expression in CEO-treated HCT116 cells, compared to untreated control cells, indicating a significant decrease in cell proliferation. Studies have indicated that CEO effectively inhibited tumor cell proliferation by suppressing the expression of the nuclear proliferation marker Ki67, which was in line with our results. Additionally, a significant reduction in Ki67 expression following CEO treatment, compared to control groups, was demonstrated in in vivo models of rat mammary carcinoma and Ehrlich ascites carcinoma [13,48].

Cyclins are essential regulators of the cell cycle, a family of proteins that manage the cell’s progression by activating cyclin-dependent kinases (CDKs). The levels of cyclins fluctuate throughout the various stages of the cell cycle, and their precise expression is crucial for the accurate timing and smooth transition between phases [49,50]. Cyclin D forms complexes with CDK4 and CDK6, which are key in facilitating the shift from the G1 phase to the S phase by preparing the cell for DNA replication. Cyclin D is often considered a responder to external mitogenic signals and plays a significant role in determining whether a cell should enter the division cycle [51]. The regulated expression of Cyclin D is crucial for proper cell-cycle control, and disruptions in its expression are frequently observed in various cancers [52]. The results showed that the CEO significantly decreased the percentage of Cyclin D^+^ cells, indicating a reduction in the number of cells progressing from the G1 phase to the S phase. This selective downregulation of Cyclin D pointed out that the CEO specifically interferes with cell-cycle progression at the G1 phase, potentially inducing cell-cycle arrest and inhibiting further proliferation of HCT116 cells. Nagle et al. [53] demonstrated that cinnamaldehyde, the primary active component of cinnamon essential oil, induced cell-cycle arrest at the G2/M phase in HCT116 colon cancer cells. Similarly, a study on cinnamon extract, also rich in cinnamaldehyde, reported a dose-dependent decrease in the G1 phase, accompanied by an increase in the sub-G1 population or G2/M phase arrest in HCT116 cells [41]. Furthermore, CEO treatment led to a delayed accumulation of MDA-MB-231 cells in the G0/G1 phase, along with an increase in the S phase of the cell cycle [13].

The p21, p27, and phospho-AKT (p-AKT) play crucial roles in regulating the cell cycle. The p21 and p27, cyclin-dependent kinase (CDK) inhibitors, regulate cell-cycle control by blocking the activity of CDK–Cyclin complexes, including those involving Cyclin D. These proteins can be activated in response to various stimuli, such as DNA damage or other stressors, serving as regulatory factors that arrest cell-cycle progression, particularly at the G1 checkpoint, by inhibiting the Cyclin D/CDK4/6 complex [54,55]. This inhibition is critical for the cell’s response to damage, preventing the replication of damaged DNA [55]. In contrast, p-AKT, the phosphorylated form of protein kinase B (AKT), is a key oncogenic factor involved in regulating cell proliferation and survival. The phosphorylation of AKT activates downstream pathways that promote cell growth and survival, making it a crucial target for cancer therapy. Dysregulation of AKT signaling is commonly observed in various cancers; it contributes to tumorigenesis and resistance to treatment [56]. Treatment with CEO induced significant alterations in the expression of key cell-cycle regulators in HCT116 cells. Specifically, there was an increase in the proportion of cells expressing the tumor suppressors p21 and p27. This upregulation likely contributed to CDK suppression, leading to cell-cycle arrest. Consistent with these findings, the increased expression of p21 and p27 was associated with a reduction in cyclin D levels, further supporting the disruption of cell-cycle progression. Moreover, CEO treatment significantly decreased the levels of phosphorylated AKT (p-AKT). The reduction in p-AKT, a crucial component of the oncogenic signaling pathways that support cell survival and proliferation, might be connected with a potential antitumor effect of the CEO. These changes in p21, p27 and p-AKT expression emphasized the CEO’s ability to disrupt key processes involved in cell-cycle regulation and survival signaling, highlighting its potential as an effective anticancer agent. Li et al. [57] proposed that the mechanism of action of cinnamaldehyde involved the modulation of genes associated with apoptosis, invasion, and adhesion, primarily through the inhibition of the PI3K/Akt signaling pathway.

### 3.7. In Vitro, CEO Reduces the Concentration of Pro-Inflammatory Cytokines

Generally, essential oils have been found to suppress inflammation and reduce cytokine production by disrupting key components of inflammatory pathways [38]. Furthermore, the literature indicates the effects of essential oils on the production of interleukins, tumor necrosis factor, thromboxane, and leukotrienes [58]. The evaluation of the immunomodulatory effects of CEO in this research showed that treatment with ConA only, a poly-clonal T cell activator, notably elevated the levels of IL-1β, TNF-α, IFN-γ, IL-17, and IL-10 in the cell supernatant after 24 h, compared to untreated cells [59]. Also, CEO by itself significantly raised the concentrations of IL-1β, TNF-α, IFN-γ, IL-17, and IL-10, compared to splenocytes incubated in medium. However, the combination of the ConA and CEO treatments significantly reduced the levels of IL-1β, TNF-α, IFN-γ, IL-17, and IL-10, compared to ConA stimulation alone. Furthermore, the CEO appeared to promote a predominance of pro-inflammatory cytokines over IL-10. This effect was more pronounced in non-ConA primed cells, which represented likely components of the innate immune response. Interestingly, while CEO exerted an immunosuppressive effect on activated cells, it simultaneously activated naïve cells, demonstrating a dual impact on immune-cell function. To the best of our knowledge, only a limited number of studies have investigated the effects of CEO on the production of inflammatory biomarkers. Cinnamon essential oil has been shown to significantly reduce the expression of several pro-inflammatory mediators, including vascular cell adhesion molecule-1 (VCAM-1), intercellular adhesion molecule-1 (ICAM-1), monocyte chemoattractant protein-1 (MCP-1), interferon gamma-induced protein 10 (IP-10), interferon-inducible T-cell alpha chemoattractant (I-TAC), and monokine induced by gamma interferon (MIG) in a human skin disease model [60]. Additionally, CEO suppressed IL-2 secretion in the Caco-2 human intestinal epithelial cell model and reduced TNF-α levels in lipopolysaccharide (LPS)-induced mice [42,44].

## 4. Materials and Methods

### 4.1. Essential Oil Material

CEO was purchased from Fares, Timisoara, Romania. According to the manufacturer, the essential oil was obtained by steam distillation from the bark of the species *Cinnamomum zeylanicum* Blume. The sample was stored in the original package, a dark glass bottle, at room temperature and away from sunlight before the analysis.

### 4.2. GC and GC–MS Analysis of the CEO

The chemical composition of the cinnamon bark *C. zeylanicum* essential oil was determined by gas chromatography–mass spectrometry (GC–MS) using a Shimadzu GCMSQP2010 ultra mass spectrometer (Shimadzu Corporation, Kyoto, Japan) fitted with a flame ionic detector coupled with a GC2010 gas chromatograph (Shimadzu Corporation, Kyoto, Japan). The InertCap5 capillary column (60.0 m × 0.25 mm × 0.25 µm) was employed for separation. Helium (He) was used as a carrier gas at a split ratio 1:5 and a linear velocity of 35.2 cm/s. The ion source temperature was 200 °C, the injector temperature was 250 °C, and the detector temperature 300 °C, while the column temperature was linearly programmed from 40 to 260 °C, at the rate of 4 °C/min; then from 260 to 310, at a rate 10 °C/min; and after reaching 310 °C, kept isothermally for 10 min. TPEO was dissolved in EtOH (*v*/*v* 96%) and consecutively injected in the amount of 1 µL. The contents of different compounds were determined based on the normalized area of chromatograms and defined as each content according to the corresponding peak area (the mean of three determinations). The identification of the constituents was performed by comparing their mass spectra and retention indices (RIs) with those obtained from authentic samples and/or listed in the NIST/Wiley mass-spectra libraries, using different types of search (PBM/NIST/AMDIS) and available data in the literature [61].

### 4.3. Antioxidant Activity

#### 4.3.1. DPPH Radical Scavenging Activity Assay

The antioxidant activity of CEO was evaluated by a DPPH radical (2,2-diphenyl-1-picrylhydrazyl) scavenging activity assay, which was performed according to the method described by Blois, with some modifications [62]. The reaction mixture consisted of 20 µL of an ethanolic solution of essential oil in increasing concentrations and 280 mL of 0.1 mM ethanolic solution of DPPH (0.04 mg/mL). After the 30 min’ incubation at room temperature and in a dark place, the absorbance values of the reaction mixtures were determined spectrophotometrically at 517 nm, using the BioTek Epoch 2 Microplate Spectrophotometer (Agilent Technologies, Santa Clara, CA, USA). Butylated hydroxytoluene (BHT) was used as a positive control, while pure ethanol served as a negative control. The antioxidant activity, expressed as the percentage of inhibition, was determined according to the following equation:

$$DPPH(\%)=[(A_{\mathrm{control}}-A_{\mathrm{sample}})/A_{\mathrm{control}}]\times 100$$

where A_control_ is the absorbance of the negative control and A_sample_ is the absorbance of the sample.

The results were expressed as an IC_50_ value (the sample concentration able to neutralise 50% of the DPPH free radicals), using a linear regression method.

All the measurements were performed in triplicate, and the results are expressed as the mean values for the concentration expressed as mg/mL ± standard deviation.

#### 4.3.2. Cupric Ion Reducing Antioxidant Capacity Assay (CUPRAC Assay)

The CUPRAC assay was conducted using the procedure described by Milošević et al. [63]. A quantity of 60 µL of CEO in increasing concentrations diluted in ethanol was added to a microplate in a series of wells. This was followed by the addition to each well of 240 µL of a mixture consisting of 75 µL of aqueous solution of CuCl_2_·2H_2_O (1.7 µg/mL), 75 µL of methanolic solution of neocuproine (7.5 mM), and 90 µL of ammonium acetate buffer (pH ≈ 7). The reaction mixture was incubated for 30 min at an ambient temperature. The absorbance was recorded at 450 nm using a BioTek Epoch 2 Microplate Spectrophotometer (Agilent Technologies, Santa Clara, CA, USA), with measurements conducted in triplicate. The same procedure was conducted with BHT as a positive control. The reducing activity was calculated using the following equation:

$$(\%)\;reducing\;power=[(A_{\mathrm{sample}}-A_{\mathrm{control}})/A_{\mathrm{sample}}]\times 100$$

where A_sample_ is the absorbance of the sample, and A_control_ is the absorbance of the negative control (20 μL of pure ethanol). The results are expressed as IC_50_ values, using a linear regression method. The IC_50_ values were calculated as the concentration (mg/mL) which provides 50% of the reducing power. All the measurements were performed in triplicate, and the results are expressed as the mean value ± standard deviation.

#### 4.3.3. ABTS Radical Scavenging Activity Assay

A 2,2′-azino-bis-(3-ethylbenzothiazoline-6-sulfonic acid) (ABTS) radical cation decolorization assay was applied to determine the antioxidant activity of the CEO. The assay was performed according to the method previously described by Čutović et al., with some modifications [64]. The ABTS solution was prepared by dissolving 20 mg ABTS^•+^ radical cation (7.8 mmol/L) in 5 mL deionized water; this was followed by the addition of 88 μL of aqueous solution of potassium persulfate. The incubation of the mixture lasted 16–20 h at 4 °C in the dark. The control solution was the mixture of 280 μL ABTS^•+^ radical cation solution and 20 μL of ethanol. The reaction mixture consisted of 20 μL of the sample, and 280 μL of the ABTS solution was added into microplates and incubated for 6 min at 25 ± 5 °C in the dark. The absorbance was recorded at 734 nm using a BioTek Epoch 2 Microplate Spectrophotometer (Agilent Technologies, Santa Clara, CA, USA), with measurements conducted in triplicate. The percentage of inhibition was calculated using the following equation:

$$Inhibition(\%)=[(A_{\mathrm{control}}-A_{\mathrm{sample}})/A_{\mathrm{control}}]\times 100$$

where A_control_ is the absorbance of the negative control (pure ethanol) and A_sample_ is the absorbance of the sample. The obtained results were transformed using a linear regression method. The IC_50_ values were calculated as the concentration (mg/mL) that provides 50% of the inhibition.

#### 4.3.4. Total Antioxidant Capacity Assay (TAC)

The total antioxidant capacity assay was carried out according to a modified method, based on the reduction of Mo(VI) to Mo(V) previously described by Milošević et al. [63]. An aliquot of 20 μL of essential oil dissolved in ethanol was combined with 200 μL of reagent solution (0.6 M sulfuric acid, 28 mM sodium phosphate, and 4 mM ammonium molybdate). The tubes with reaction mixture were incubated for 90 min at 60 °C. For the blank, 100 μL of deionized water was used in place of the sample. After the samples were cooled at room temperature, the absorbance of each sample was measured at 695 nm in the spectrophotometer. The calibration curve was made with L-ascorbic acid solution as a positive control (0.3125–10 mmol/L). The results are expressed as L-Ascorbic Acid Equivalents (mg AAE/g) of three replicates.

### 4.4. Antimicrobial Activity

#### 4.4.1. Microbial Strains

Standard strains of microorganisms sourced from the American Type Culture Collection (ATCC, Microbiologics, St. Cloud, MN, USA) were utilized in this research. The antimicrobial activity of the essential oil was tested on seven bacterial strains and one yeast strain. The Gram-positive bacteria tested were *S. aureus* (ATCC 6538), *S. epidermidis* (ATCC 1228), and *B. subtilis* (ATCC 6633). The Gram-negative bacteria included *E. coli* (ATCC 8739), *P. aeruginosa* (ATCC 9027), *K. pneumoniae* (ATCC 13883), and *S. enterica* serovar Abony (ATCC 6017). Moreover, the antimicrobial effect was evaluated on *C. albicans* (ATCC 10231) as a yeast strain.

#### 4.4.2. Determination of Minimum Inhibitory Concentration (MIC)

The minimum inhibitory concentration (MIC) of the essential oil was determined using the broth microdilution method in standard sterile 96-well flat-bottomed microtiter plates according to the Clinical and Laboratory Standards Institute guidelines [15]. Fresh microbial cultures were established by incubation on Müller–Hinton agar (TSA; Torlak, Belgrade, Serbia) for 24 h at 37 °C. Individual colonies were then suspended in saline solution to a density of approximately 1.5 × 10^8^ CFU/mL and measured using a DEN-1 McFarland densitometer (Biosan, Riga, Latvia). Bacterial suspensions and *C. albicans* yeast were diluted with Müller–Hinton broth to final concentrations of 1.5 × 10^6^ CFU/mL for bacteria and 2 × 10^6^ CFU/mL for *C. albicans*, and 100 µL of the suspension was added per well. Dilutions of CEO were prepared by dissolving it in dimethyl sulfoxide (DMSO); this was followed by serial dilutions in broth. These dilutions were added in equal volumes to each well, yielding final concentrations between 7.37 and 118 μg/mL. The microtiter plates were incubated at 37 °C for 24 h. Each concentration was tested in duplicate and the broth microdilution test was repeated three times. The standard antibiotics minocycline and amikacin, as broad-spectrum antibiotics commonly used in clinical practice for the treatment of various types of Gram-positive and Gram-negative bacterial infections, along with the antifungal agent nystatin, were used as positive controls. The results are expressed as minimum inhibitory concentrations (MIC)—the lowest concentrations of the tested essential oil at which no microbial growth is observed. MIC values indicate bacteriostatic and fungistatic activities.

### 4.5. Cytotoxic Activity

#### 4.5.1. Cell Culture

In this study, a variety of cell lines were carefully cultured, including mouse (4T1) and human (MDA-MB-468) breast carcinoma cells, mouse (CT26) and human (HCT116) colon carcinoma cells, and a human fibroblast cell line (MRC-5). These cell lines were obtained from the American Type Culture Collection (ATCC), Microbiologics, St. Cloud, MN, USA. All cell lines were cultured in Dulbecco’s modified Eagle’s medium (DMEM) supplemented with 10% fetal bovine serum (FBS) from Sigma Aldrich, St. Louis, MO, USA. The cultures were maintained in a controlled environment with 5% CO_2_ under standard laboratory conditions. For the experiments, only cell suspensions with over 95% viability were used. Cell viability and counts were carefully determined using trypan blue staining, ensuring the accuracy and reliability of the experimental outcomes.

#### 4.5.2. In Vitro Evaluation of the Cytotoxic Effects of CEO

To assess the cytotoxic activity of CEO, an MTT (3-(4,5-dimethylthiazol-2-yl)-2,5-diphenyltetrazolium bromide) assay was performed, in line with previously established protocols [65,66]. The MRC5, 4T1, MDA-MB 468, CT26, and HCT116 cell lines, all in the exponential growth phase, were harvested from culture flasks, counted, and seeded into 96-well plates at a density of 5 × 10^3^ cells per well. After allowing 24 h for cell adhesion, 100 μL of serially diluted CEO and cisplatin (ranging from 0.78 to 100 μg) were added to each well. The cells were incubated for 48 h at 37 °C in a 5% CO_2_ incubator. Following incubation, the plates were centrifuged, the supernatant was removed, and 100 μL of MTT solution (5 mg/mL in PBS) was added to each well for a 4 h incubation. The plates were then centrifuged again, the cell-free supernatants were discarded, and 150 μL of DMSO, along with 20 µL glycine buffer, was added to dissolve the formazan crystals. After shaking the plates for 10 min, the optical density of each well was measured at 595 nm using a Zenyth 3100 microplate reader (Anthos Labtec Instruments GmbH, Salzburg, Austria). The percentage of cytotoxicity was calculated in accordance with the procedures described in a previous study [43].

### 4.6. Evaluation of Apoptosis

To evaluate apoptosis in HCT116 cells treated with CEO, the Annexin V and propidium iodide double staining assay was used, following protocols from previous studies [39,64]. This method enabled the quantification of cells in early and late apoptosis, as well as those undergoing necrosis. In additional experiments, cells were fixed and permeabilized with a permeabilization buffer from BD Bioscience, Heidelberg, Germany. They were then incubated with antibodies specific to Bcl-2, Bax, and caspase-3, following previously established procedures [43]; these antibodies were provided by Thermo Fisher Scientific Inc., Waltham, MA, USA. The treated cells were analyzed using a FACS Calibur flow cytometer (BD Biosciences, San Jose, CA, USA), and the data were processed with FlowJo software (v10.8.2). This method allowed for a detailed and comprehensive evaluation of the apoptotic responses in cells exposed to the various treatments, providing deeper insight into the apoptotic mechanisms activated under the experimental conditions.

### 4.7. The Impact of CEO on Cell-Cycle Dynamics

The potential effects of the CEO on the cell-cycle dynamics of HCT116 tumor cells were further investigated. Specifically, the Ki67 expression in tumor cells treated with the CEO was evaluated and compared to untreated cells. The experimental setup included a treatment group exposed to IC_50_ concentrations of the CEO, and a control group of untreated cells; all were cultured in 25 mL flasks. After a 24 h incubation post-treatment, the cells were trypsinized, washed three times with PBS, and counted. Each sample was then stained with Ki67-specific antibodies (eBioscience, San Diego, CA, USA) and 1 μL of Vybrant^®^ DyeCycleTM Ruby stain (Thermo Fisher Scientific, Inc.). The samples were analyzed using a FACS Calibur flow cytometer (BD Biosciences, San Jose, CA, USA), with at least 15,000 events per sample being analyzed. Data analysis was performed using FlowJo vX.0.7 software, providing a detailed evaluation of the cell-cycle phases.

### 4.8. Analysis of the Effects of CEO on Cell-Cycle Regulators

HCT116 tumor cells were treated with the IC_50_ concentration of the CEO or maintained in culture medium alone as a control for 24 h, after which they underwent a series of cellular analyses. The cells were first fixed and permeabilized using a permeabilization buffer from BD Bioscience, Heidelberg, Germany. They were then incubated with specific antibodies targeting key cell-cycle regulators, including Cyclin D, p21, p27, and phospho-AKT (p-AKT), all supplied by Thermo Fisher Scientific, followed by secondary FITC-conjugated Dnk anti-rabbit IgG mAb (Abcam Limited, Cambridge, UK). The cells were analyzed using a FACS Calibur flow cytometer (BD Biosciences, San Jose, CA, USA), and the data were processed and interpreted using FlowJo software (v10.8.2).

### 4.9. In Vitro Assessment of the Immunomodulatory Impacts of CEO

The impact of CEO on cytokine production by splenocytes was assessed. Splenocytes from healthy BALB/C mice were isolated as per a previously established method [67]. Cell viability was evaluated using a trypan blue exclusion assay, and only those with over 95% viability were used for subsequent experiments. The splenocytes were placed in 96-well plates (1 × 10^5^ cells per well) and divided into four groups: a control group incubated with complete medium, cells stimulated with concanavalin A (ConA) (0.5 µg/mL), cells treated with CEO (IC_50_), and cells treated with both CEO and ConA. The cells were incubated for 24 h at 37 °C in a humidified environment with 5% CO_2_. After incubation, cell viability was confirmed using the trypan blue assay, showing that viability was above 90% in all groups, with no significant differences. The cells were then centrifuged, and the supernatants were stored at −80 °C for later analysis. Cytokine levels of IL-1β, TNF-α, IFN-γ, IL-17, and IL-10 were quantified using high-sensitivity ELISA kits from R&D Systems (Minneapolis, MN, USA) as specified by the manufacturer [68].

### 4.10. Statistical Analysis

All measurements of antioxidant activity evaluation were taken in triplicate, and the results are expressed as mean values with standard deviation (SD). Calculation of IC_50_ values was performed using Microsoft Excel 365. The antimicrobial assay involved the testing of each concentration in duplicate across three independent experiments, and the results are expressed as MIC values. The data were analyzed using SPSS software, version 26.0. Statistical comparisons were performed using the nonparametric Mann–Whitney U test and Kruskal–Wallis test. The normality of data distribution was assessed using the Kolmogorov–Smirnov test. A *p*-value of <0.05 was considered statistically significant, *p*-values of <0.01 were regarded as highly significant, and *p*-values of <0.001 were considered very highly significant.

## 5. Conclusions

The present study revealed 99.66% of the total chemical composition of commercial *C. zeylanicum* bark essential oil, with (*E*)-cinnamaldehyde being a major component (77.93%), followed by eugenol (4.34%), *E*-caryophyllene (3.68%), and linalool (2.79%). The essential oil has shown considerable antioxidant activity and strong antimicrobial activity, with a more pronounced effect on Gram-positive bacteria and *C. albicans* than on Gram-negative bacteria. In addition, the essential oil exhibited pronounced antitumor effects, particularly against HCT116 colorectal cancer cells, in which it induced apoptosis through modulation of Bax, Bcl-2, and caspase-3 expression, and triggered G1-phase cell-cycle arrest via downregulation of Cyclin D and p-AKT. The oil also significantly reduced the proliferation marker Ki67. Importantly, CEO demonstrated selectivity by sparing non-cancerous MRC-5 fibroblasts. Furthermore, the essential oil exerted immunomodulatory effects by reducing the production of pro-inflammatory cytokines (IL-1β, TNF-α, IFN-γ, and IL-17) in activated splenocytes, while enhancing pro-inflammatory responses in non-activated, naïve immune cells.

These findings highlighted the great potential of cinnamon bark essential oil in the development of new therapeutic agents.

## Figures and Tables

**Table 1:** Chemical composition of *C. zeylanicum* bark essential oil.

N°	Class/Compound	Kovats Index	Percentage (%)
Monoterpene Hydrocarbons			
1	α-thujene	924	0.06
2	α-pinene	932	0.65
3	Camphene	946	0.05
4	β-pinene	974	0.21
5	α-phellandrene	1002	0.58
6	α-terpinene	1014	0.10
7	*p*-cymene	1020	1.38
8	Limonene + β-phellandrene	1025 + 1025	1.45
9	Terpinolene	1086	0.06
Oxygenated Monoterpenes			
10	1,8-cineol	1026	0.83
11	Linalool	1095	2.79
12	Terpinen-4-ol	1174	0.30
13	α-terpineol	1186	0.58
Aromatic Compounds			
14	Benzaldehyde	952	0.09
15	Phenyl ethyl alcohol	1106	0.10
16	Eugenol	1356	4.34
17	Eugenol acetate	1521	0.11
18	Benzyl benzoate	1759	0.75
Sesquiterpenes			
19	α-copaene	1374	0.14
20	(*E*)-caryophyllene	1417	3.68
21	α-humulene	1452	0.24
22	*Cis*-muurola-4(14),5-diene	1465	0.09
23	Caryophyllene oxide	1582	0.16
Cinnamic Derivatives			
24	(*Z*)-cinnamaldehyde	1217	0.05
25	(*E*)-cinnamaldehyde	1267	77.93
26	(*E*)-cinnamylacetate	1443	2.74
27	(*E*)-allyl cinnamate	1548	0.20
Total			99.66

**Table 2:** Antioxidant activity of C. *zeylanicum* bark essential oil and a standard antioxidant, based on the DPPH, ABTS, CUPRAC, and TAC assays.

	IC_50_ DPPH (mg/mL)	IC_50_ ABTS (mg/mL)	IC_50_ CUPRAC (mg/mL)	TAC (mg AAE/g)
CEO	9.53 ± 0.20	0.35 ± 0.01	0.14 ± 0.02	0.01 ± 0.00
BHT	0.011 ± 0.001	0.006 ± 0.001	0.02 ± 0.00	9.80 ± 0.15

The results are expressed as means (n = 3) ± SD. CEO—Cinnamon essential oil; BHT—Butylated hydroxytoluene.

**Table 3:** Antimicrobial activity of CEO and standard antibiotics, expressed as MIC (µg/mL).

Bacterial Strain	ATCC	CEO MIC (µg/mL)	MIN MIC µg/mL	AMK MIC µg/mL	NYS MIC µg/mL
*Staphylococcus aureus*	6538	7.37	0.40	1.00	N.t.
*Staphylococcus epidermidis*	1228	7.37	0.25	N.t.	N.t.
*Bacillus subtilis*	6633	14.75	0.05	3.25	N.t.
*Escherichia coli*	8739	14.75	N.t.	12.00	N.t.
*Klebsiella pneumoniae*	13,883	14.75	N.t.	12.50	N.t.
*Pseudomonas aeruginosa*	9027	29.50	N.t.	15.00	N.t.
*Salmonella enterica* subsp. *enterica* serovar Abony	6017	14.75	N.t.	12.50	N.t.
**Yeast strain**					
*Candida albicans*	10,231	7.37	N.t.	N.t.	0.85

N.t.—Not tested. MIN—Minocycline; AMK—Amikacin; NYS—Nystatin.

**Table 4:** IC_50_ Values for CEO and cisplatin (CDDP) on the MRC-5, 4T1, MDA-MB 468, CT26, and HCT116 cell lines, as determined by the MTT assay.

Cell Lines	IC_50_ (µg) 48 h	
	CEO	CDDP
MRC-5	7.21 ± 0.25	4.08 ± 2.11
4T1	12.43 ± 4.55	1.33 ± 0.74
MDA-MB 468	3.78 ± 0.93	3.44 ± 1.34
CT26	<0.78	<0.78
HCT116	1.18 ± 0.93	5.16 ± 1.82

**Table 5:** Selectivity index (SI) for CEO and cisplatin (CDDP) on the MRC-5, 4T1, MDA-MB 468, CT26, and HCT116 cell lines.

Cell Lines	Selectivity Index (IC_50_ MRC-5/IC_50_)	
	CEO	CDDP
4T1	0.6	3.1
MDA-MB 468	1.9	1.2
CT26	N/A	N/A
HCT116	6.1	0.8

## Data Availability

All data generated for this study are included in the article.

## Abbreviations

The following abbreviations are used in this manuscript:

CEO	Cinnamon essential oil
GC-MS	Gas chromatography–mass spectrometry
DPPH	2,2-diphenyl-1-picrylhydrazyl
ABTS	2,2′-azino-bis-(3-ethylbenzothiazoline-6-sulfonic acid)
CUPRAC	Cupric ion reducing antioxidant capacity
TAC	Total antioxidant capacity
BHT MIC	Butylated hydroxytoluene Minimum inhibitory concentration
MIN	Minocycline
AMK	Amikacin
NYS	Nystatine
IC50	Concentration that provides 50% of inhibition
MTT	(3-(4,5-dimethylthiazol-2-yl)-2,5-diphenyltetrazolium bromide)
4T1	Mouse breast cancer cells
MDA-MB-468	Human breast cancer cell line
CT26	Mouse colon cancer cell line
HCT116	Human colon cancer cell line
MRC-5	Human fibroblast cell line
CDDP	cis-diamminedichloroplatinum(II) or cisplatin
Ki67	Marker of proliferation
p21	Cyclin-dependent kinase inhibitor 1A
p27	Cyclin-dependent kinase inhibitor 1B
p-AKT	Phosphorylated AKT
ConA	Concanavalin A
IL-1β	Interleukin 1 beta
TNF-α	Tumor necrosis factor alpha
IFN-γ	Interferon gamma
IL-17	Interleukin 17
IL-10	Interleukin 10
